# The Nursing Supplement

**Published:** 1888-09-29

**Authors:** 


### The Nursing Mirror.

All Contributions for this Supplement should be addressed "The Nursing Mirror," care of The Editor, 38, Old Jewry, E.C.

### En Passant.

A LESSON ON PHYSIOLOGY.—A professor, when taking the physiological class of Mrs. ———'s ladies' seminary, was explaining the theory that the body is entirely changed every seven years, and addressing Miss Jones, said: "There you see, Miss Jones, you will no longer be Miss Jones seven years hence." Shy Miss Jones cast down her eyes and said, "I hope so."

THE FATAL MISTAKE AT BELFAST.—Miss Torrens (who so unluckily administered crude carbolic acid instead of black draught to a patient) is progressing favourably, and is now out of danger. An unusual number of members have attended the last meetings of the board of the Royal Belfast Hospital, and the melancholy occurrence has been fully discussed. The adjourned inquest will shortly take place, and Nurse Torrens may probably have to be sent to trial on a charge of manslaughter.

THE NURSING OF OVARIOTOMY.—There used to be an old-fashioned idea that in certain serious abdominal operations the patient should be left in charge of one nurse for a space of forty-eight hours. There seems to be some doubt whether certain surgeons do not still cling to this mistaken notion, so we would point out its great cruelty both to patient and nurse. It is absolutely impossible that any woman could give unbroken attention to one point for two days and two nights without the slightest rest or change; the idea is preposterous. Think how little any person is good for after the loss of one night's sleep, and then contemplate the state of the woman who is in charge of a critical case and is yet denied her natural rest? She cannot do justice to her patient; she must do damage to her own health. Then what pride and vanity it fosters in a nurse if she be allowed to think that she alone, and no other, can attend to a patient at the most critical period? Where nurses are thoroughly trained to observe and to report intelligently on what they see, there is not the slightest reason why there should not be a day and a night nurse for a serious case, the one quite capable of always taking up and carrying on the thread of observation and care where the other drops it. It shows bad management, bad training, and selfishness and vanity, when one nurse cannot trust another to take her place. It was the fault which marred the grand character of Sister Dora that she liked to have all authority and responsibility, and delegate no duties of any consequence to anyone else; so that when she was absent everything fell into confusion, and her vanity was gratified by feeling that she was missed. We publish a letter this week on the subject of the nursing of ovariotomy.

A TRUE STORY.—With regard to the nursing of small-pox, a doctor tells the following story, from which nurses may draw several morals: "An old friend of mine was taken ill with confluent small-pox several years since, and I attended him. It was a very bad case indeed, and I got three nurses to watch him, that they might never get exhausted, but always give their keenest attention to their duties. I also telegraphed for a well-known physician, who was a specialist with regard to fevers; when he saw my patient he said there was no hope, my old friend would die on the eighth day of the disease. That was the fourth day, and in spite of this verdict I visited my patient regularly thrice a day, at ten, two, and ten, and bade the nurses not give up hope but continue to do their best. The eighth day arrived, and I visited my patient twice, and he seemed certainly past aid. After dinner I felt very anxious about him, and as nine o'clock rose to go to him again. My wife pointed out that it was an hour earlier than usual, and bade me wait for my coffee, but I declined, and walked down to the house, which was not far off. When I entered the room all three nurses were there, one with a folded napkin in her hand. 'Is he dead?' I asked. 'Yes, sir; he died about three minutes ago.' I went forward and could see that the jaw had dropped, but further than this it was impossible to distinguish any feature of the face, which was covered completely with exudation about a-quarter of an inch thick. I put my hand to the left side and thought I felt a slight flutter; immediately I put my arm behind the patient and raised him, and putting my finger well into the open mouth, swept it round and cleared it of the exudation of which it was full. Just like a clock which has stopped, and when shaken goes on, my old friend began to breathe again. I stayed by his side all night, cleaning out the mouth at intervals, and he breathed regularly. You who know how unpleasant is the odour of small-pox, even under the best conditions of nursing, can imagine that this was a tiring night after a long day's work. My friend recovered, and the point I want you specially to notice is that had I not gone to visit him rather earlier than usual, the nurse would have tied up the mouth and smothered the last flicker of life that remained."

ANOTHER TRUE STORY.—An amusing anecdote of a dream is told in a paper called *On the Road.* The teller, Miss Rose S. MacBride, of the Foundling Hospital, started in the Scotch express, and fell asleep: "Suddenly there was a terrible crash and confusion, and all the horrors of a railway accident were upon us. I struggled to extricate myself from the *débris*, but I could not move without pain: surely my legs were broken. How long I lay there I could not say; but at last a gentleman and one of the porters came to my assistance. The doctor, for such he was, examined my limbs and ordered someone to go for a stretcher. Upon this I was laid and carried off to the hospital. I did not move or speak; I closed my eyes and lay like a log. I must at this time have lost my senses, for when I opened my eyes again I was on the table in the operating theatre. Several doctors came in and looked at me, and one of them muttered 'double amputation immediately.' How awful it seemed to me no one can imagine, I seemed deprived of all power of speech and only gazed before me in dumb stupidity. I watched the nurses flitting from one place to another; they lit the fire, opened cupboards, and brought out large bottles, basins, sponges, and trays full of knives, also a basket containing lint, bandages, and other things used at operations. One nurse sprinkled the floor with sawdust, another lit a lamp and put it under a brass thing that soon reminded me of a small steam engine hissing forth a quantity of strong-smelling steam. At last everything seemed ready for the slaughter, and I wondered at myself for taking things so quietly. The ghastly-looking knives and saw were laid upon the table, and I watched the surgeon tie a large apron round his waist; he then rolled up his sleeves and prepared for action. A bag was put over my mouth, and I was told to 'breathe like a good girl.' I felt that my end had come: who can guess the agony of that moment! I felt as if I was smothering, and made a last effort to scream. I must have been pretty successful, for I felt myself roughly shaken by the arm, and heard a woman's voice saying 'Why, bless the girl, she has frightened me nearly to death: she has got the nightmare, surely.' Imagine my joy when I realized it was all a horrible dream."

### Letters from a Probationer.

No. IV.—Throughout the Night.

Dear ——, I have been put on to night duty in a women's surgical ward, and find the change very restful and pleasant. You know I had a perfect horror of night work before I came, but I have felt so tired out in body lately that I was only too glad to be appointed to work which involved less standing and running about. The first night seemed very long and gloomy, but I was not a bit sleepy, and I sat and read a good deal. The gas is turned low, and there is only a small screened lamp for the nurse—a little speck of light in the long dark ward. There is a senior nurse in the next ward, to whom I have to refer should anything go wrong, and the night superintendent comes round at intervals. The first two nights were so quiet I began to grumble at the dulness of my post, but the third night I had an awful experience. There was an amputation case I had special orders to watch and keep near, and about three o'clock I noticed that hæmorrhage had set in. I had been shown where to compress the femoral artery, and I immediately pressed my two thumbs on the place, while I called in a low voice to the girl in the next bed. She was semi-convalescent, and so could run and tell the senior nurse without risk. It seemed ages while I made her understand what I wanted, and when she was gone someone at the far end of the ward began to groan, and call out "Nurse!" I called back in a low voice, "Be quiet, Ten; I will come directly." The nurse just came in swiftly for a moment to see what had occurred, and that I had temporarily arrested the bleeding, and then she went off for the house-surgeon. Meanwhile, I was standing over my patient, pressing with my two thumbs with all my might—doubtless with far more force than was necessary. The surgeon, night superintendent, and nurse all came into the ward together, and the surgeon said curtly, "Hold on a minute longer," while he hastily undid the dressing. I was in despair, and almost said I *could* not press any longer, but the habit of obedience was strong, and I did my best. Does it strike you as absurdly weak that I should have found a quarter-of-an-hour in one position so trying? Well, before you laugh at me you just attempt it yourself; it is no joke, I can assure you. At last I was released, and the night superintendent, seeing I was just done for, bade me go to the passage window for awhile. Oh, how beautifully calm and peaceful the skies looked after that awful little episode in the ward! I sat on the window seat beside the open window and let the gentle night breeze blow against my face. The town was so dark and still, and up in the deep, dark heaven thousands of placid stars were keeping watch over this feverish world. Simply to gaze at them in their steady brightness gave me strength and tranquillity, and soon I was able to go back to my post, not fearing to face the remainder of the night. Since then I have discovered that it is a rare thing for a night to pass quietly without some excitement, and often the hours of darkness are as busy as those of the day. I had a dreadful scene only last night with a half-drunken woman, who had a dislocated shoulder. As a rule such a slight case is not admitted to the wards; but the surgeon was in a kindly mood, and sent word that the woman was to be got quietly to bed, and then he would come and see to her. She soon fell into a heavy sleep, and then the surgeon came along and sat down on the side of the bed. He asked for a towel, which he put in the armpit under the dislocated shoulder; then he put his foot up against the towel, and catching hold of the woman's arm with both his hands, he gave it a sudden wrench and back it flew into the socket. The woman gave a groan and half woke up and cursed a little, but was soon asleep again. The surgeon strolled quietly away, and this morning the woman was discharged, having slept off her drunkenness. Now I must go away to bed, though it is broad daylight, and you are just sitting down to lunch.

### Everybody's Opinion.

[*Correspondence on all subjects is invited. No communications can be entertained if the name and address of the correspondent is not given, or unless one side of the paper only be written on.*]

### A NURSE'S POSITION.

It is evidently a fact that trained nurses are becoming more essential and indispensable every day. Thanks to the good discipline and delicacy of our hospital training, the nurse is not only considered competent to nurse, but has sufficient influence over her patients to command that respect which is due to her womanly dignity, where even their relatives or friends fail.

A Reader of "Overheard in the Train."

### NURSING OVARIOTOMY CASES.

Having just nursed a lady through this serious operation, and seen her walk downstairs at the end of four weeks, I offer these hints, which may be useful to young nurses. The reason why doctors are so particular about the choice and arrangement of a sick-room is because the patient must spend all the time in the room, and unless a large amount of fresh air is let in the patient must suffer. Care, too, should be taken that all the foul air of the house does not ventilate into the bedroom. The following is a list of things which every nurse should know: Room for operation should be well ventilated, and kept about 63 to 65 degs. Less furniture the better; no carpets or curtains. Room to be well washed with carbolic and well sprayed with carbolic for some hours before the operation; no food for three hours before. Bowels well cleared with enema; catheter used before being placed on the table. Lower limbs clothed in warm woollen clothing, and flannel jacket put on. The patient should be well washed with carbolic a day before, and the abdomen a few hours before. It is well to have ready a large waterproof sheet, with hole in centre and a strip of plaster sewn round the margin of the hole. Sponges should be well soaked in carbolic, one in 20 or one in 40. Lotion, brandy, ice, hot and cold water, and plenty of towels at hand. After the operation, patient should be in every sense at rest. In case of sickness the hand should be placed with pressure over side of wound; on no account should the patient be allowed to move. No food should be given without doctor's orders; a little iced water in case of thirst would be a comfort to the patient. Urine to be measured and temperature taken. A diet chart should be kept for the doctor. Nurse L. H.

### THE QUESTION OF COURAGE.

Whilst your correspondent "M." was to some extent in error in saying that a nurse runs no risk of contracting small-pox if she has been successfully re-vaccinated, every one who knows anything of the nature of small-pox must admit that the re-vaccinated cases who fail with it are very few, and those few only suffer from the disease in the most modified form—three or four spots forming the sum total of the rash. The sisters and attendants whom you mention as failing with small-pox at Manchester, I conclude, had it in a mild form, and if only re-vaccinated after the disease was discovered in their midst, there can be no doubt that the virus had entered the system prior to re-vaccination. Inasmuch as after successful re-vaccination it is impossible to have more than a mild attack of small-pox (and very improbable even that), I contend that the risk we run is reduced to a minimum. I quite agree with you that typhoid is only contagious, but, to insure our own safety when nursing it, one must take certain precautions, all of which would be quite useless in small-pox without the only real safeguard of re-vaccination. The cases of typhus in this country may not be very numerous: nevertheless, a nurse on the staff of an infectious hospital must hold herself prepared to nurse it, and so also must the private nurse who has engaged to do infectious work for the institution to which she is attached; and there is as much danger from one or two as from one or two dozen cases. We cannot gather from the remarks whether "M." is a nurse or not, or whether the opinion expressed has been arrived at by personal experience or otherwise. For myself, I speak from some years of experience, both in London and the provinces. T. A.

### VOLUNTEER NURSES.

IN your issue of the 11th ult., I notice a suggestion about the enrolment of volunteer nurses. I for one would like to have my name on the roll of volunteer nurses, and gladly sacrifice my time and labour should an invasion ever occur. If anything is done in this matter, I suppose it will be published in your paper, and then one will know how to make application. THE HOSPITAL seems to increase in interest. I do not know what I should do without it. It seems to carry one back, on perusal each week, to one's London Hospital days—some of them the happiest in my life.

Philadelphia, September 10th. PHILOPENA.

### Appointments.

[It is requested that successful canddates will send a copy of their applications and testimonials, with date of election, to THE EDITOR, The Lodge, Porchester Square, W.]

JENNY LIND INFIRMARY.—Miss Wainwright has been appointed matron of the Jenny Lind Infirmary for Sick Children, Norwich, in place of Miss Peter, who has resigned after three and a-half years of excellent work. Fifty-seven candidates sent in testimonials; but these had been reduced, after careful consideration by a sub-committee, to three or four, and these ladies attended on September 10th to afford the committee the advantage of a personal interview. After the interview, at which the qualifications of the candidates received careful consideration, a vote was taken, the result of which was that Miss Wainwright was declared duly elected as the new Lady Superintendent. Miss Wainwright's testimonials are of the highest order; she was trained at the well-known Children's Hospital, Pendlebury, where she was for two years and eight months, and has consequently had great experience in the general treatment of children. She is also a highly-trained nurse, having been for two years as staff nurse in St. Bartholomew's Hospital, London, and also as sister at the East London Hospital for Children, Shadwell, for a year, and is therefore well-fitted to have lady probationers and nurses under her.

GREAT YARMOUTH NEW HOSPITAL.—Miss A. M. Whitfield, late of the Northern Hospital, Liverpool, has been appointed the first matron of the New Hospital at Great Yarmouth. Miss Whitfield was trained at Addenbrooke's, and possesses many excellent testimonials.

WEST KENT HOSPITAL.—Miss Isabel Jones has been appointed Lady Superintendent of this hospital. She was trained at Guy's, charge Sister Dora Convalescent Hospital, and Sister at the Leicester Infirmary. There were 63 applicants, cf whom eight were seen by the committee.

MISS MARY WALKER has been appointed sister-in-charge of the Nurse's Home of the London Hospital, in place of Miss Freeth, resigned.

IN the American Civil War, between the Northern and Southern States it was found that nothing was better for the sick and wounded, winter and summer, than a tent or a ridge ventilated hut. Erysipelas and hospital gangrene were almost unknown; wounds healed more rapidly; and even in fractures the beneficial effects of the fresh air were to be remarked.

### Lectures.

THE prospectus of the Toynbee Hall autumn session is now out, and contains the following announcements which may interest nurses:—

On October 8th and following Mondays, a course of ten lectures on "The Chemistry of the Arts and Manufactures" will be given by Vivian B. Lewes; commencing at eight p.m. Fee 1*s*.

On October 12th and following Fridays, a course of ten lectures on "Physiology" will be given by Walter Pye, F.R.C.S. Two reading parties will be connected with this course. Fee 1*s*.

On November 3rd, at eight p.m., Miss Jane E. Harrison will lecture on "Hospitals among the Greeks," with magic lantern illustrations.

On November 24th, at eight p.m., Mrs. Fawcett will lecture on "Men and Women." Admission by tickets, to be obtained from the sub-warden.

The Sunday afternoon oratorio services at St. Nicholas Cole Abbey have begun again; commencing at a-quarter to four.

To day, at a-quarter to five, is the harvest festival service at the Hospital for Sick Children, Great Ormond Street.

On October 25th a meeting will be held at Mrs. R. Poole's, 5, Beaumont Street, Oxford, to discuss the question of the registration of midwives.

On October 2nd, 3rd, 4th, and 5th, a course of lectures will be delivered at Gresham College, by the Gresham Professor of Medicine; to commence at six p.m. Admission free.

### Vacancies.

The following vacancies are announced:—

**Matrons.**

Nurses' Home, Newcastle-on-Tyne.

Barnet Cottage Hospital; £30.

**Night Superintendent.**

Hospital for Sick Children, Pendlebury.

**Nurses.**

Throat Hospital, Golden Square.

Sidcup Cottage Hospital; £26

Bradford Infirmary; £25

County Hospital, Guildford; £20.

Birkdale Nurses' Home, East Southport.

Brompton Hospital for Consumption.

Sheffield Infirmary; several; £21.

Nurses' Institute, Canterbury.

Nursing Institute, Northampton.

Wrexham Infirmary; £25

Royal Alexandra Children's Hospital, Brighton; £20.

Nottingham and Notts Nursing Institution; Several.

Stockton Hospital; £20.

Homes for Aged Mariners, Egremont, Cheshire; £25.

Royal National Hospital for Consumption, Ventnor; £20.

General Hospital, Tunbridge Wells; £25.

Blackheath Institute for Trained Nurses; £20.

Nursing Institution, Cheltenham; Several.

**District Nurses.**

Exmouth. Keighley. Wilton.

**Probationers.**

Lloyd Cottage Hospital, Bridlington.

National Hospital for the Paralysed and Epileptic.

Royal Hospital for Diseases of the Chest.

Buckinghamshire General Infirmary, Aylesbury.

### Notes and Queries.

TO CORRESPONDENTS.—1. Questions may be written on post-cards. 2. Advertisements in disguise are inadmissible. 3. In answering a query please quote the number. 4. If a private answer is desired, a stamped addressed envelope must be forwarded.

**Queries.**

(63) *Massage.*—Nurse Maud would be much obliged if the editor would give her information through the "Nursing Mirror's" Queries where a thorough training in massage can be obtained in London and Edinburgh. Nurse Maud has been told of a Dr. Dowse, in London, who trains pupils in massage, but does not know if that is the case.

(65) Where can nurses be sent for change and disinfection after nursing fever cases?—*Louise Smith.*

**Answers.**

(64.) *Nurse Private.*—You could gain no certificate of nursing by attending lectures, which would be of use professionally. There are the St. John's Ambulance lectures on first aid to the wounded, and on nursing, both of which would increase your knowledge and give you certificates, but those certificates would gain you no position in a hospital. No hospital grants a certificate save to those who have worked for years within its walls.

(63.) *Massage.*—The hospital of which Dr. Dowse is physician is in Welbeck Street, and training is there given in massage. Miss Hall, 242, Oxford Street, also teaches massage.

(62) C. O. will be taken as a probationer at the Hospital for Women and Children, Cork, Ireland, where she will get first-class training.—*Eva Scott.*

(65) Nurses should send all clothing worn in the sick-room to a proper disinfecting oven, and should themselves take a carbolic bath, being sure to cleanse the hair thoroughly. After putting on perfectly fresh clothing throughout, they can go anywhere for a change, keeping as much as possible in the open air.

